# Examining affective structure in chickens: valence, intensity, persistence and generalization measured using a Conditioned Place Preference Test

**DOI:** 10.1016/j.applanim.2018.07.007

**Published:** 2018-10

**Authors:** Elizabeth S. Paul, Joanne L. Edgar, Gina Caplen, Christine J. Nicol

**Affiliations:** Bristol Veterinary School, University of Bristol, Langford House, Langford, Bristol, BS40 5DU, UK

**Keywords:** Chickens, Hens, Preference, Emotion, Affect, Valence

## Abstract

When measuring animals’ valenced behavioural responses to stimuli, the Conditioned Place Preference (CPP) test goes a step further than many approach-based and avoidance-based tests by establishing whether a learned preference for, or aversion to, the location in which the stimulus was encountered can be generated. We designed a novel, four-chambered CPP test to extend the capability of the usual CPP paradigm to provide information on four key features of animals’ affective responses: valence, scale, persistence and generalization. Using this test, we investigated the affective responses of domestic chickens (*Gallus gallus domesticus*) to four potentially aversive stimuli: 1. Puffs of air; 2. Sight of (robotic) snake; 3. Sprays of water; 4. Sound of conspecific alarm calls. We found conditioned avoidance of locations associated with the air puffs and water sprays (Friedman’s χ^2^_(3)_ = 13.323 p > .005; χ^2^_(3)_ = 14.235 p > .005), but not with the snake and alarm calls. The scale of the learned avoidance was similar for the air puff and water spray stimuli, but persistence and generalization differed. We conclude that the four chambered CPP test can have a valuable role to play in making multi-feature measurements of stimulus-generated affective responses, and we highlight the value of such measurements for improving our understanding of the structure of affect in chickens and other animals.

## Highlights

• Conditioned Place Preference tests were used to study affective responses in hens.
• Both air puff and water spray stimuli produced conditioned place aversions.
• A robotic snake, and the sound of conspecific alarm calls, did not.
• Negative affective responses can differ in their persistence and generalization.

## 1 Introduction

Decades of research have revealed many of the preferences of domestic laying hens and broilers (*Gallus gallus domesticus*) for different environments and resources and their motivation to gain access to these, and this information has been invaluable for the development and design of housing and husbandry systems to improve welfare (e.g. Cooper and Appleby, 2003; Dawkins, 1983; Hughes and Black, 1973; Nicol, 1986; Olsson and Keeling, 2002). Less studied to date, however, are these birds’ affective responses to the variety of discrete stimuli and events that they may encounter during their daily lives, many of which may be aversive to them (e.g. Bertolus et al., 2015; Cooper et al., 1998; Pajor et al., 2000; Rutter and Duncan, 1992). For example, on farm, including free-range farms, chickens can experience a range of potentially punishing events (i.e. events that they would avoid if possible), including the sudden onset of loud noises or bright lights, flickering lights, rain, wind and encounters with aggressive conspecifics or predators (e.g. Kristensen et al., 2007; McAdie et al., 1993). In recent years a number of emotion theorists have proposed that animals’ long-term affective states or “moods” represent integrations of both the reward and punishment experiences of their day-to-day lives, not only as a result of encountering preferred and non-preferred resources and environments, but also from their experiences of more briefly encountered, discrete stimuli and events such as those listed above (Eldar et al., 2016; Mendl et al., 2010; Nettle and Bateson, 2012). If this is correct, it is important to find out whether and to what extent different, short-term events and stimuli are perceived by animals as being punishing or rewarding, as each of these events may contribute significantly to their long-term affective states and welfare. The experiments reported in this paper consider this issue for chickens in particular.

In addition to immediate concern for the welfare of farmed chickens, these animals’ evaluations of potentially punishing stimuli and events are valuable to study because of the role such information can play in furthering our knowledge and understanding of the affective states of these animals and how they are structured. For example, it is well known that many animals, including chickens, find the taste of quinine aversive, as evidenced by their behavioural responses to ingesting it and by its capacity to act as an instrumental punisher (e.g. see Dwyer, 2011; Sherwin et al., 2002). And this knowledge can be used in the design of experiments that investigate the multiple components of negative affective responses, and their effects on learning and behavioural decision-making (e.g. Harlander-Matauschek et al., 2009; Steiner et al., 2001). However, our understanding of the many other sorts of discrete stimuli that domestic chickens experience as punishing is far from complete, with the consequence that researchers are sometimes left having to make guesses and assumptions about the aversiveness of particular stimuli, rather than basing their studies on empirical evidence. For example, in a study of anticipatory behaviour, Zimmerman et al. (2011) proposed the explicit assumption that sprays of water would be perceived as aversive (negative) by hens.

### 1.1 The structure of affective states

Despite a dramatic increase in research interest in the topic of animal affect in recent years (for examples of recent reviews see: Bliss-Moreau, 2017; Gygax, 2017, Paul & Mendl in press, Perry and Baciadonna, 2017; Weary et al., 2017), important questions remain about the structure and function of affective states in a wide range of species, including birds such as the domestic chicken. Punishing or aversive stimuli can vary in their severity, frequency, and the nature of their consequences. For example, a negative affective state can be produced by a physical stimulus that has a direct effect on the animal, whether that is a severe injury or a brief disruption of physiological homeostasis. A negative affective state might also arise from exposure to stimuli that have meaning for the animal (e.g. visual or auditory stimuli that predict the advance of a predator), yet have no direct physical effects. Whether and how animals’ responses to these types of stimuli differ, and how their consequent affective responses might vary, is not well understood. Certainly, some punishers are stronger and have more intense effects than others. But it is also possible that the affective consequences of different types of punisher differ in ways that go beyond strength or intensity. For example, some punishers may have mild yet long lasting effects, while others might have a powerful but only brief impact. In other words, the structure of the resulting affective states may vary according to more than one dimension, and different types of punisher may influence these dimensions differentially.

Anderson and Adolphs (2014) proposed an influential framework for studying the multi-faceted structure of affective states and responses in a wide range of non-human animals. They suggested that many animal vertebrates and even some invertebrate species can be shown to possess four “emotion primitives” – basic building blocks of what we call “emotion” in humans. They argued that in addition to the two commonly discussed dimensions of affect, “valence” (positivity vs negativity of response) and “scalability” (magnitude or intensity of response), two further properties, “persistence” and “generalization”, should also be regarded as defining features of affective (emotion-like) states in animals. Persistence represents the extent to which affective responses endure over time following their initial triggering. Examples of this in humans are commonplace, with states such as anxiety and depression sometimes long out-lasting the event or events that triggered them (e.g. see Charney et al., 1998). But Anderson and Adolphs (2014) point out that this sort of behavioural and physiological persistence of response can also be seen in a wide variety of animal species. For example, pigs exposed to brief bouts of social isolation, restraint and loud noise while away from their home pens show reduced activity levels once returned to their home pens (Reimert et al., 2017). And even in *Drosophila*, noxious air puffs promote a persistent, elevated motor activity (Lebestky et al., 2009). Generalization concerns the tendency for stimuli similar to a primary emotive stimulus to have a capacity to arouse equivalent (albeit often less intense) affective responses in a likewise manner. This fourth feature of affective responses can also be seen in a range of animals, both in the form of generalized instrumental and classically conditioned responses (e.g. in rodents – McLaren and Mackintosh, 2002), and more recently in judgement bias tests in which affective state manipulations are seen to influence subject animals’ responses to novel and ambiguous stimuli (e.g. Harding et al., 2004; Mendl et al., 2009).

It is possible to conduct a range of behavioural tests to assess a variety of aspects of both the valence and the scale of an animal’s response to a stimulus and thereby to establish whether, and how much of, a positive or negative state has been induced. Such tests include approach-avoidance tests, preference tests, consumer demand tests, cognitive bias tests and progressive ratio tests (e.g. Dawkins, 1990; Duncan, 1978; Harding et al., 2004; Hodos, 1961; Mendl et al., 2009). Tests for the persistence and generalization of affective responses are less common, however (Anderson and Adolphs, 2014), although the process of generalization has been the subject of research in the field of animal-human (stock person) interactions for a number of years (e.g. see Brajon et al., 2015; Breuer et al., 2003). To better understand how different types of stimuli differentially and interactively influence all four of these “emotion primitives”, an experimental approach is needed which is able to assess all of these facets of affect within a single, unified paradigm. We propose that a modified version of a conditioned place preference test has utility in this regard.

### 1.2 The Conditioned Place Preference Test

The method that was developed for use in the present experiments to assess the affective valence, scale, persistence and generalization of domestic chickens’ responses to a range of potential punishers was the Conditioned Place Preference (CPP) Test (also sometimes known as the Conditioned Place Aversion Test when punishers are studied – e.g. Wang et al., 2017). CPP Tests were originally designed and used within the discipline of psychopharmacology and have been employed extensively to investigate the psycho-affective properties of a range of drugs including opiates, benzodiazepines and selective serotonin reuptake inhibitors (for reviews see e.g. Bardo and Bevins, 2000; Tzschentke, 2007). They are based on the principle of classical conditioning and the observation that many animals readily develop conditioned associations between the features of a location (e.g. in distinctively coloured or patterned chambers of an experimental testing box) and the discrete stimuli that they experience while there. In most CPP experiments, a two-chambered apparatus is used, in which one chamber or compartment of a test box is paired with a stimulus (e.g. provision of a food or injection of morphine) while the adjacent compartment is paired either with no stimulus, or a sham control (e.g. injection of saline). When subsequently given the choice to spend time in the chamber that was previously paired with the stimulus, or the one that was not, an animal’s preference for the stimulus-paired location is interpreted as an indication that the original, unconditioned stimulus had been perceived by the animal to be relatively rewarding (indicating positive affective valence), or vice versa in the case of a punishing stimulus (indicating negative affective valence). To avoid possible confounds resulting from animals that have pre-existing preferences for the coloured or patterned location cues (i.e. the discriminative stimuli, which should ideally be affectively neutral themselves), associative pairings are generally counterbalanced between subjects, and the outcome measures used are based, not on absolute preferences, but on changes in preference occurring between the pre- and post-conditioning phases.

Although CPP tests have predominantly been employed for neurological and psycho-pharmacological research in rodents (see Tzschentke, 2007), they have also had some use in farm animals in recent years (e.g. de Jonge et al., 2008), including chickens and chicks (Buckley et al., 2012; Dixon et al., 2013; Jones et al., 2012; Nasr et al., 2013). In the present experiments, we sought to make use of the CPP paradigm to find out whether four potentially punishing stimuli can be said to generate negative affective states in chickens, and to attempt to extend the usefulness of the CPP test making measurements of all four facets of affective responses outlined by Anderson and Adolphs (2014) Traditionally, the CPP test is used to measure the relative valence of an animal’s affective response to a stimulus and its control, and a measure of scale or intensity can also be obtained from the proportional amount of time spent by the animal in the stimulus-associated, as opposed to non-stimulus-associated chamber following conditioning. In the present experiment, we also tested for three consecutive days post-conditioning, to obtain a further measure of the temporal persistence of any affective response. And by employing a novel, four-chambered apparatus design, with not only stimulus-associated and neutral (non-stimulus associated control) chambers, but also chambers adjacent to these, which shared colour but not pattern as discriminative stimuli, it was also possible to investigate whether any of the affective responses investigated varied in terms of their capacity to generalize – that is, to be generated by stimuli similar but not identical to the original associative stimulus.

### 1.3 Using the CPP Test to measure negative affect in chickens

The experiments presented here were designed to investigate laying hens’ affective responses to four potentially punishing, negatively valenced, stimuli. We hypothesised that the birds would learn to avoid the neutral environmental cues of the CPP apparatus (discriminative stimuli) associated with each stimulus type. The stimuli were chosen to be suitable for use in an experimental situation, yet also to bear a resemblance to the sorts of potentially punishing events that might be encountered by domestic hens in their everyday lives, and hence have relevance for hen welfare. For ethical reasons, the potential punishers were also chosen to be relatively mild in their effects, inflicting no actual physical harm on the birds. The stimuli investigated in Experiment 1 were brief puffs of air (designed to mimic windy or draughty conditions on a farm), and the sight of a potential “predator” (a robotic snake). In Experiment 2, we assessed hens’ responses to the sound of conspecific alarm calls (tape playbacks), and brief sprays of water (from a plant-misting device, designed to mimic showers of rain for free ranging hens).

We hypothesised that the sight of a snake and the sound of alarm calls, although not in themselves hazardous, may represent evolved reinforcers that are perceived negatively by birds because of their ancestral value in predicting predatory attacks, and hence may have affective consequences for modern hens (similar examples of associative, “primary reinforcers” include the sight of angry faces and snakes in modern humans; e.g. see Mallan et al., 2013). The puff of air and spray of water were different in that they had direct physical effects on the birds; we hypothesized that although these are not likely to be regarded as being threatening in the sense of resembling an ancestral prediction of predation, they would nevertheless generate negatively valenced responses, to the extent that cumulative experiences of cooling air and water can have significantly detrimental effects, both to hens’ energetic states and feather conditions.

## 2 Methods and materials

### 2.1 Ethical statement

The experiments were carried out under Home Office project licence number 30/2779, and the hens were re-homed to private, free-range, small-holdings at the end of testing.

### 2.2 Animals and housing

The research took place in a University of Bristol research animal building. Subject animals were 48 commercially bred hens (ISA Warrens) obtained from a local stockist at approximately 20 weeks of age. All birds were group-housed in indoor pens (302 cm × 363 cm), 8 birds per pen, 12:12hr light-dark cycle, 18–22 °C with *ad libitum* food and water, perches, nest boxes and dust-bath available.

### 2.3 Experimental design

Three consecutive batches of hens were brought into the research facility for training and testing for Experiment 1 (n = 8 per batch), and two consecutive batches were brought in for Experiment 2 (n = 12 per batch). Upon arrival, each bird was randomly assigned to an experimental group, according to the stimulus to which they were to be exposed: Experiment 1, Group 1. Air puff (n = 12); Group 2. Robotic Snake (n = 12); Experiment 2, Group 1. Water Spray (n = 12), Group 2. Conspecific Alarm Calls (n = 12). Birds from each batch were assigned in equal numbers to each experimental group.

### 2.4 Equipment

**Fig. 1:**
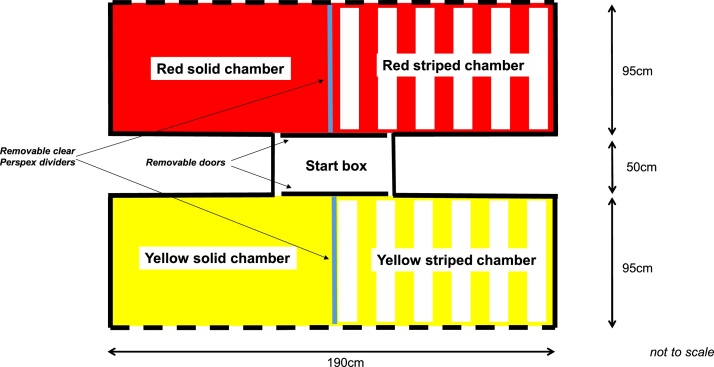
Four-chambered CPP apparatus, viewed from above (not to scale).

The Conditioned Place Preference (CPP) apparatus was constructed of plywood, with total dimensions 190c x 240 cm, and a height of 60 cm. It consisted of a central start box with removable doors which provided access to both sides of the apparatus. The interior walls and floor of one side of the apparatus were painted red, and the other side yellow. Each side was further partitioned into two chambers, separated by a clear Perspex removable divider, and painted either in solid colour (red, yellow) or striped in the same colour with 6 cm stripes (see Fig. 1).

The outer walls of each side of the apparatus were made of chicken-wire, through which the test stimuli could be encountered: Air puffs (Experiment 1, Group 1) were directed through the chicken-wire walls, via a 60 cm length of flexible plastic tubing, using a manually controlled spray-duster cannister (Invertible Gas Duster, *AF Sprayduster*). The Robotic Snake (Experiment 1, Group 2) was a remote controlled, plastic child’s toy, 2 cm wide and 60 cm long, and decorated with horizontal brown, pale orange and black bands across the length of its body. It was placed on the outside the apparatus, approximately 5 cm distance from the chicken-wire wall and moved slowly parallel to the outer wall. The apparatus used to apply the Water Spray stimuli (Experiment 2, Group 1) to the hens was a pump-trigger plant sprayer (Karcher), with rigid plastic lance. The conspecific Alarm Calls (Experiment 2, Group 2) were recorded from a separate group of birds and played back using a solid-state audio recorder (Marantz PMD 661 MK II; Hampshire, UK) at 50 dB.

### 2.5 Procedure

The experiments took place over the course of three weeks, comprising for each bird, an initial week for habituation, a second week for baseline (pre-conditioning) preference testing and conditioning, and a third week for post-conditioning preference testing.

#### 2.5.1 Week 1: habituation

Across four consecutive days, hens were habituated to being handled, and to the rooms and equipment in which the testing would be carried out. This involved repeated 10-minute sessions in which the hens were exposed to human presence in the home pen, handling in the home pen, removal from home pen to the adjacent experimental room, and placement in the start box of the CPP apparatus (with doors in place so that the rest of the apparatus was not visible), until no escape attempts were observed and the birds moved quietly and confidently.

#### 2.5.2 Week 2: Baseline place preference testing and conditioning

On the morning of the first day of Week 2, all birds were given separate, 10-minute experiences of each side of the CPP apparatus (Red side or Yellow side first, order randomly counterbalanced within experimental groups), with full access to both the solid and striped areas within each coloured side (i.e. central Perspex dividers not present). In the afternoon, they were given 10-minute free access to the whole of the CPP apparatus (i.e. Red side and Yellow side), starting in the Start Box (no dividers or doors in place).

On the second day, baseline preference testing took place: Each hen was placed individually in the start box and given free-choice access to all areas of the apparatus for 10 min, by removing all doors and dividers. The location of the hen within the apparatus was continuously observed and timed using a stopwatch, with the total duration spent in each of the five locations (Start box, Red solid chamber, Red stripe chamber, Yellow solid chamber, Yellow stripe chamber) being recorded.

**Fig. 2:**
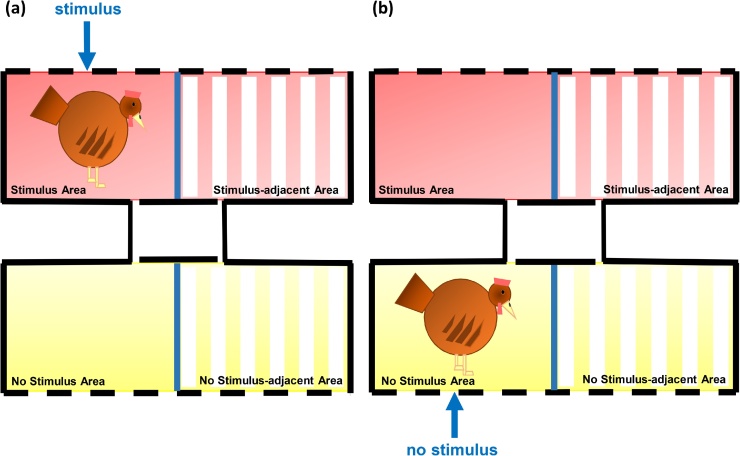
Conditioning phase of Experiments 1 and 2: (a) Hens were exposed to the stimulus for three 5-minute conditioning trials in the “Stimulus Area” of the apparatus, and (b) Hens were also exposed to no stimulus for three 5-minute conditioning trials in the “No Stimulus Area”. The colour and pattern of these areas were counter-balanced between birds.

Across the following three days, conditioning took place. The hens were exposed to six, five-minute conditioning sessions (2 per day, at least 90 min apart) in which a particular area of the apparatus (“Stimulus Area”, e.g. Red striped chamber) was associated with presentation of the experimental stimulus (Air Puff or Robotic Snake), while the pattern-matched area on the other side (“No Stimulus Area”, e.g. Yellow Striped chamber) was associated with no stimulus presentation (see Fig. 2). Each hen experienced one stimulus session and one no-stimulus session per day (in randomised order, alternating each day), and the same colour/pattern - stimulus associations were maintained across the three days.

To perform a conditioning session, all dividers and doors were in place so that the bird remained in just one chamber of the CPP apparatus. Each hen was placed individually into the chamber she had been assigned for conditioning session, and a wire mesh lid placed overhead. When the condition was “No Stimulus”, no stimuli were presented; when the condition was “Stimulus”, stimuli were presented, starting 30 s after the bird was placed in the chamber. If the stimulus was “Air Puff” (Experiment 1, Group 1), two brief bursts of air were directed towards the bird using the spray canister at 30 s intervals. If the stimulus was “Robotic Snake” (Experiment 1, Group 2), the toy snake moved slowly beside the outside edge of the chamber, visible to the hen through chicken-wire walls. If the stimulus was “Water Spray” (Experiment 2, Group 1), a plant sprayer was used to deposit two fine jets of water onto the back of the test hen at 30 s intervals. The bird was then dried prior to being returned to the home pen after the end of the 5-minute training period. If the stimulus was “Alarm Call” (Experiment 2, Group 2), a pre-recorded sound file (comprising alarm calls recorded from hens unfamiliar to the test group) was played to the test bird in 30 s bursts of audio at 30 s intervals (starting at 30 s).

At the end of these five-minute conditioning sessions, the birds were returned to their home pens. Any water, faeces, etc. on the floor of the test apparatus was removed prior to the training of the next bird.

#### 2.5.3 Week 3: conditioned place preference testing

Following conditioning, the place preferences of each hen was determined, during 10-minute testing sessions on each day for three consecutive days. These tests were conducted in the same way as the baseline preference tests (see above). Up to 90 min. prior to testing on each day, hens were exposed to two further conditioning sessions (as above, order alternating), refreshing the associations between the stimulus/no stimulus and the relevant colour/pattern pairings of the chamber.

### 2.6 Data and analyses

Initial analyses were conducted to assess whether subject birds showed any preferences for the colour and pattern stimuli used as discriminative stimuli within the CPP apparatus, and to establish whether the hens showed any changes in the amount of time spent in the start box across the course of the experiment. Total duration data for time spent by subject hens in each chamber of the CPP apparatus were obtained for the four separate testing sessions: Baseline (pre-conditioning); Test Day 1, Test Day 2, Test Day 3 (post-conditioning). The percentage of time spent during these ten-minute tests in each of the four chambers of the apparatus (excluding time spent in the start box) was then calculated for all birds and all test sessions, providing four data points per hen per test: Percentage of test time spent in Stimulus Area, Stimulus-adjacent Area, No Stimulus Area and No Stimulus-adjacent Area. These non-independent variables were taken as markers of the learned relative attractiveness or aversiveness each of the four areas. Significant changes in time spent in the Stimulus Area between Pre-conditioning and post-conditioning tests were interpreted as a valenced response to the stimulus having occurred, with the magnitude of this change being taken as a measure of the Scale (intensity) of this response. Persistence was indicated by whether any changes in time spent occurring on post-conditioning Test Day 1 also continued, on Test Days 2 and 3. And Generalization was inferred by the pattern of chamber-use changes that took place between pre- and post-conditioning tests. Specifically, we anticipated that if a stimulus generated a generalizable, negative affective response, the hens would learn an aversion to both the Stimulus Area and the Stimulus-Adjacent Area, which shared a discriminative cue (colour of walls and floor), and would redirect their use of the chambers of the CPP apparatus towards spending more time in the No-Stimulus adjacent area, which shared neither discriminative cue (colour nor pattern) with the Stimulus Area.

Because these time-spent data were not normally distributed (they were left skewed), and transformations (square root, log and reverse score) were not successful in normalising them, non-parametric analyses were used throughout. Friedman tests were used to examine within-subject changes in the percentage of time spent in each of the four areas of the CPP apparatus across the four testing sessions (Baseline, Test Day 1, Test Day 2, Test Day 3), with post-hoc Wilcoxon signed-rank tests being used to make pairwise comparisons between the Baseline Test and each of the three post-conditioning Test days when Friedman tests were significant. Because of technical problems, one bird from the Air Puff condition (Experiment 1, Group 1) and two birds from Robotic Snake condition (Experiment 1, Group 2) were not able complete Test Day 3, reducing sample sizes for these pairwise comparisons to n = 11 and n = 10 respectively.

## 3 Results

### 3.1 Experiment 1: Effects of exposure to air puffs and a robotic snake on the conditioned place preference test

#### 3.1.1 Baseline preferences

Across all 24 birds in this experiment, binomial tests revealed no significant preferences at Baseline for spending time either in the Red or Yellow side of the CPP apparatus nor for Striped or Solid patterned areas, although Group 1 birds (that went on to experience conditioning with the Air Puff stimulus) showed a significant preference for the Striped pattern, with the birds spending a median of 65.20% of their time in these areas (p < .05). Birds in Group 2 (that went on to experience the Robotic Snake stimulus) did not show this preference.

#### 3.1.2 Start Box durations

Friedman analyses showed that the total time spent by Group 1 and Group 2 birds in the start box of the apparatus did not vary significantly between Baseline testing and Test days 1, 2 and 3.

#### 3.1.3 Group 1: air puff stimulus

**Fig. 3:**
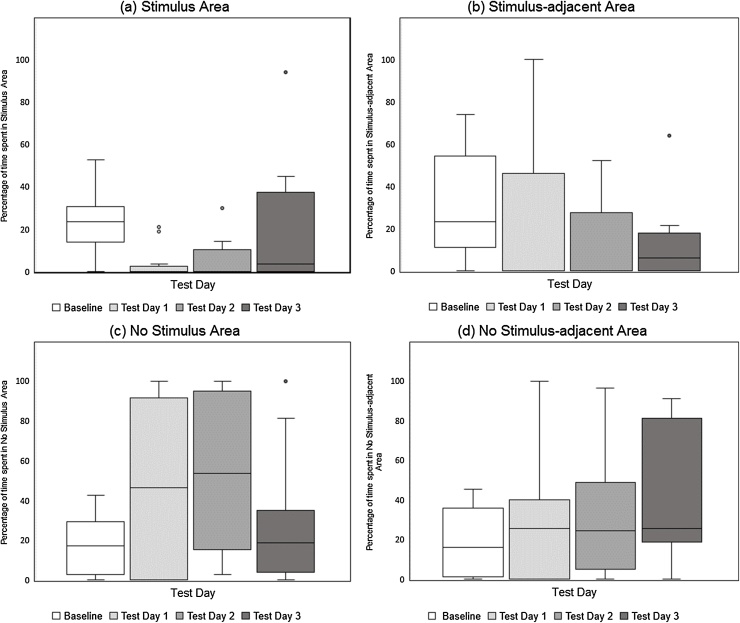
Experiment 1, Air Puff Stimulus. Box and whisker plots of percentage of 10-minute test sessions spent by hens in each of the four areas of the CPP apparatus: (a) Stimulus Area, (b) Stimulus-adjacent Area, (c) No Stimulus Area, (d) No Stimulus-adjacent Area.

Changes in the percentage of time spent by hens in all four areas of the CPP apparatus between Baseline and the three post-conditioning tests are illustrated in Fig. 3(a–d). A Friedman’s test demonstrated that the percentage of time the hens spent in the Stimulus Area of the CPP apparatus (in which they had experienced Air Puff during conditioning) varied significantly across the four test sessions (Baseline, Test Day 1, Test Day 2, Test Day 3), χ^2^_(3)_ = 13.323 p < .005. Pre-planned Wilcoxon signed-rank tests were conducted to establish whether Stimulus Area use changed between the Baseline test and post-conditioning Tests 1, 2 and 3 (i.e. three comparisons). Using a Bonferroni correction (i.e. these three tests required a minimum significance level of p < .0167 for equivalence with p < .05 for a single test), the percentage of time the birds spent in the Stimulus Area was found to be significantly lower on Test Days 1 and 2 than on the Baseline Testing Day (p = .006 and p = .008 respectively). Birds reduced the amount of time they spent in the Stimulus Area by a median of 19.42% between Baseline and Test Day 1, and 18.27% between Baseline and Test Day 2. The percentage of time spent in the Stimulus Area did not differ between Baseline and Test Day 3.

Friedman tests also revealed variation across test sessions in the time spent by birds in the Stimulus-adjacent Area of the CPP apparatus (the area immediately adjacent to the one in which the they had experienced Air Puff), χ^2^_(3)_ = 8.515 p < .05. Wilcoxon signed rank tests revealed that the percentage of time spent in the Stimulus-adjacent Area was significantly lower on Test Day 3 than at Baseline (a median drop of 9.17%), but that Baseline and Test Days 1 and 2 did not differ.

The percentage of time spent by hens in the No Stimulus and No Stimulus-adjacent Areas did not vary significantly across the four test sessions.

#### 3.1.4 Group 2: robotic snake stimulus

The percentage of time the hens spent in the Stimulus, Stimulus-adjacent, No Stimulus and No Stimulus-adjacent Areas of the CPP apparatus did not vary significantly across the four test sessions.

### 3.2 Experiment 2: Effects of exposure to water sprays and conspecific alarm calls on the conditioned place preference test

#### 3.2.1 Baseline preferences

Binomial tests revealed no significant preferences at Baseline for spending time either in the Red or Yellow side of the CPP apparatus nor in the Striped or Solid patterned chambers, either for all 24 subject birds together, or for each experimental group separately (Group 1, Water Spray conditioned birds and Group 2, Alarm Call conditioned birds).

#### 3.2.2 Start Box durations

Friedman analyses showed that the total time spent by Group 1 and Group 2 birds in the start box of the apparatus did not vary significantly between Baseline testing and Test days 1, 2 and 3.

#### 3.2.3 Group 1: water spray stimulus

**Fig. 4:**
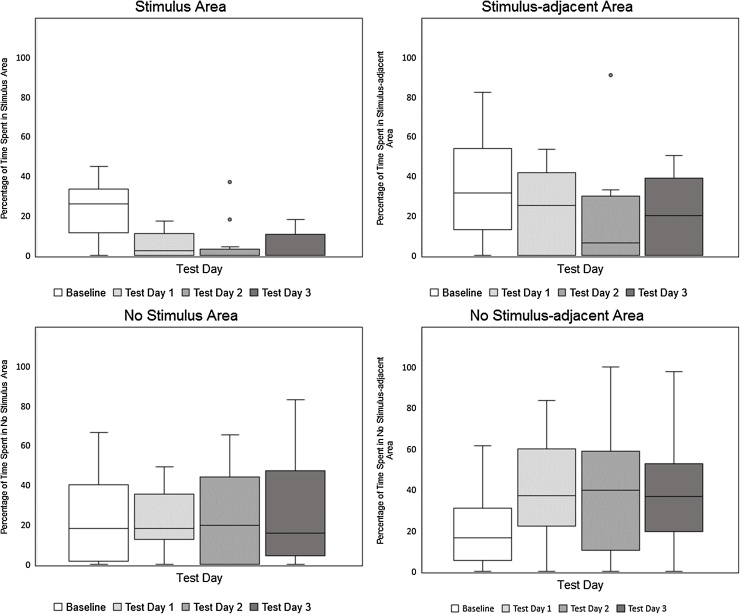
Experiment 2, Water Spray Stimulus. Box and whisker plots of percentage of 10-minute test sessions spent by hens in each of the four areas of the CPP apparatus: (a) Stimulus Area, (b) Stimulus-adjacent Area, (c) No Stimulus Area, (d) No Stimulus-adjacent Area.

Changes in the percentage of time spent by hens in all four areas of the CPP apparatus between Baseline and the three post-conditioning tests are illustrated in Fig. 4(a–d). A Friedman’s test demonstrated that the percentage of time the hens spent in the Stimulus Area of the CPP apparatus (in which they had experienced the Water Spray during conditioning) varied significantly across the four test sessions, χ^2^_(3)_ = 14.235 p < .005. Pre-planned Wilcoxon signed-rank tests, with Bonferroni correction for multiple comparisons, demonstrated that the percentage of time the birds spent in the Stimulus Area was significantly lower on Test Days 1, 2 and 3 than on the Baseline Testing Day (p = .004, p = .008, p = .008 respectively). Birds reduced the amount of time they spent in the Stimulus Area by medians of 20.16% between Baseline and Test Day 1, 17.94% between Baseline and Test Day 2, and 21.27% between Baseline and Test Day 3.

The percentage of time the hens spent in the Stimulus-adjacent and No Stimulus Areas of the CPP apparatus did not vary significantly across the four test sessions Baseline, Test 1,2 and 3. Percentage of time spent in the No Stimulus-adjacent Area, however, did vary across test sessions, χ^2^_(3)_ = 9.873, p < .05. Wilcoxon signed rank tests, with Bonferroni correction revealed that No Stimulus-adjacent Area use increased significantly between Baseline and Test Day 1 (p = .008, a median increase of 17.89%), but not significantly between Baseline and Test Days 2 and 3.

#### 3.2.4 Group 2: alarm call stimulus

The percentage of time the hens spent in the Stimulus Area of the CPP apparatus reached near-significance in its variation across the four test sessions, χ^2^_(3)_ = 7.455, p = .059, but Wilcoxon signed rank tests revealed no significant differences between Baseline and Tests Days 1, 2 and 3. The percentage of time the hens spent in the Stimulus-adjacent, No Stimulus and No Stimulus-adjacent Areas of the CPP apparatus did not vary significantly across the four test sessions Baseline, Test 1, 2 and 3.

## 4 Discussion

The present experiments were designed to investigate laying hens’ responses to four different stimuli that we hypothesised would be mildly punishing. Making use of a novel, four-chambered conditioned place preference (CPP) test, our dual aims were to (1) establish whether these four stimuli could be said to generate negatively valenced affective states in hens, and (2) to assess the additional dimensions of these affective responses, in particular to find out whether their scale, persistence and generalization (Anderson and Adolphs, 2014) covaried in a consistent pattern, or differed according to the triggering stimulus.

### 4.1 Water spray and air puff induce negatively valenced affective responses

Two of the four stimuli tested here significantly influenced subject hens’ place preferences: Air Puff (Experiment 1) and Water Spray (Experiment 2). Birds that experienced the Water Spray and the Air Puff during conditioning sessions showed subsequent shifts in their use of the four areas of the CPP apparatus, spending reduced amounts of time in the Stimulus Area (i.e. the chamber in which the Air Puff orWater Spray had been experienced) during testing sessions following conditioning. We interpret these findings as evidence that both the Air Puff and Water Spray stimuli produced negative affective states in subject birds, in the sense that they gave rise to associative avoidance behaviour. That is, although these types of stimuli have no harmful physical effects, and appear mild and transient in the behaviour they induce in subject hens (brief head shaking and feather ruffling), they nevertheless generate an avoidance response that is available to associative conditioning (i.e. that can be associated with location cues within the CPP apparatus). These findings are consistent with the use of both these stimuli as punishers in previous experimental studies (e.g. see Davies et al., 2015; Deakin et al., 2016; Edgar et al., 2012, 2013; Zimmerman et al., 2011), and also consistent with findings from other species such as rats (e.g. see Browning et al., 2017). We conclude that air puff and water spray stimuli are good candidates for future studies of affective structures and processes in hens, especially given their transient and benign physical effects. They are also likely to be good candidates for future studies of the longer-term effects that repeated experiences of aversive stimuli may have on hen behaviour and welfare (Mendl et al., 2010).

### 4.2 Sound of alarm calls and sight of snake – no evidence of negative affective responses

Our findings here that the two stimuli that had direct effects on the hens (Air Puff and Water Spray) generated conditioned avoidance behaviour in the CPP test, while the Alarm Calls and Robotic Snake did not, have a number of potential explanations. First, it is possible that any affective responses that the birds had to these latter stimuli were simply milder and/or more variable in their effects than those generated by the Air Puff and Water Spray, and so not detectable in the test as used here (i.e. a quantitative difference). Such quantitative differences may have arisen for a number of reasons, including the relative strength of the stimuli used here (e.g. air puffs delivered at a lower pressure could have produced lesser responses and alarm calls delivered at higher volumes could have produced greater ones), and the developmental experiences of the experimental hens (e.g. the present hens could have been sensitized to air puffs, or air-puff-like stimuli in an earlier part of their lives, but desensitized to conspecific alarm calls).

Second, a qualitative difference may have been detected by the CPP test. We had hypothesised that the sight of a snake-like robot and the sound of conspecific alarm calls, although not in themselves hazardous, may represent evolved (primary) reinforcers that stimulate negative affective responses in birds because of their ancestral value in predicting injury or death. The puff of air and spray of water were different in that they had immediate physical effects on the birds (which could potentially induce feather damage or disruption of thermoregulation). It is possible, therefore, that while primary reinforcers that signal threat but do not deliver actual harm can induce reflexive or other stimulus-driven behaviours (Rangel et al., 2008), they may not always produce “valenced affective states” in the sense of generating a response in which the reinforcement value of the stimulus is available to learning. That is, they may trigger immediate behavioural responses of withdrawal or approach in hens, but these behaviours are not in themselves rewarding or punishing and therefore not detectible within the CPP test. Findings that chicks readily approach mother hens’ food calls (e.g. Fischer, 1976), but did not, in Jones et al.’s (2012) conditioned place preference study, show a preference for them, is consistent with this construction, and with a conception of animal emotion (in its broadest sense) as a multi-layered phenomenon in which behaviours acting under relatively simple stimulus-response control systems, including reflexive behaviours, operate alongside a number of more complex systems of behavioural control (e.g. see Paul & Mendl in press). However, other interpretations of the current findings also exist. For example, there may be specific constraints on learning which limit the capacity of hens to make associations between potential threat stimuli such as alarm calls and robotic snakes and the types of location cues used in CPP testing, even though other sorts of associations remain possible. If this is the case, other types of tests, such as tasks which measure hens’ willingness to work to avoid (or gain access to) such stimuli, may indicate valenced affective states being generated by these stimuli, even though the CPP test does not.

Other types of qualitative difference between birds’ affective responses to the different stimuli may also have been operating in the present experiments. For example, we already know that different kinds of preference test do not all detect the same aversions to predator-like stimuli in chickens (Browne et al., 2011); specifically, hens show lesser aversions to these stimuli if they have free access to both approach and avoid them, than if they are required to make a single choice to enter a pen with or without the stimulus present. Browne et al (2011) suggested that birds’ affective responses to such threats may be influenced by factors such as perceived control over the situation (i.e. to move about, inspect and also escape if necessary). In the testing phase of the CPP task used here, the hens could move freely around the entire apparatus, and as a result may have had reduced negative affective responses to the snake and alarm call stimuli because they possessed some element of control over the situation (see also Bassett and Buchanan-Smith, 2007). To investigate these possibilities further, future comparisons will need to be made in which these qualitative and quantitative differences can be disentangled.

An additional possible explanation for the present findings is that the Robotic Snake and Alarm Call stimuli generated states of mixed valence for the subject hens: the robotic snake may have induced a predator-inspection-like state, inducing attraction and aversion simultaneously (e.g. see Blaszcyk, 2017). Similarly, conspecific alarm calls might have produced states of both negative and positive valence, alerting birds to potential threat while at the same time reassuring them that other hens were nearby. Mixed affective responses to stimuli have been postulated to explain a number of behavioural and psychological effects, but remain a topic of debate and controversy (e.g. see Larsen et al., 2017; Russell, 2017). Further investigations of these stimuli, making use of additional methods of investigation in addition to the CPP test, may help to shed further light on this possibility.

### 4.3 Scale, persistence and generalization of negatively valenced states

The scale of the valenced responses observed here – the strength or intensity of the negatively valenced states induced by the Air Puff and Water Spray stimuli - was gauged in the present experiments by the total changes in time spent by hens in the Stimulus Area between pre-conditioning (Baseline) and post-conditioning tests (Test Days 1,2,3). For example, if an animal suffered intense negative affective states during its exposures to a stimulus and learned a strong aversion the conditioned cues as a consequence, we would expect it to spend a greater proportion of its time away from the Stimulus Area during post conditioning testing than if it had experienced only a mild negative response. Here, the Air Puff and Water Spray stimuli resulted in approximately the same reduction in Stimulus Area use, post-conditioning, by birds (a median of approximately 20% of time budget shift in both cases), suggesting a similar and relatively mild level of aversion in both cases. How this degree of aversion would calibrate against other stimuli, however, is not yet clear. For example, given that the costs of avoiding particular chambers of the CP apparatus are likely to be low (e.g. no food or other resources are forfeited), this method may have most value for measuring scale of aversion to mild and moderately punishing stimuli, but may be relatively insensitive to differences between stronger, more punishing stimuli.

As the Post-conditioning Test Days (1,2,3) progressed, we expected that a complex range of processes, including habituation and extinction, would gradually reduce birds’ avoidance of the Stimulus Area of the CPP apparatus, and this was indeed the case for birds that had experienced the Air Puff and Water Spray stimuli. But we also hypothesised that the scale and persistence of the hens’ affectively valenced responses might not always covary in an identical manner. Perhaps it is adaptive for certain conditioned avoidances to persist for longer than others even if the magnitude of the original response is similar. For example, responses to threat stimuli might be initially strong, but diminish rapidly in the absence of any actual harm (e.g. Nesse, 2005). What we found here was significant conditioned avoidance of the Stimulus Area continued for two post-conditioning days in the case of the Air Puff and for three days in the case of the Water Spray (see Fig. 2, Fig. 3), indicating slightly longer lasting effects amongst birds that had experienced the Water Spray, even though the Water Spray stimulus did not produce a stronger initial aversion. Our findings appear, therefore, to confirm the idea that persistence and intensity or scale may not always directly reflect one another, although the reason why this difference was found between the Water Spray and Air Puff stimuli, is not yet clear.

Generalization – the capacity for stimuli that are similar to an original emotive stimulus to evoke similar affective responses – is argued by Anderson and Adolphs (2014) to be a fundamental feature of affective processing, although the extent to which such generalization varies from stimulus to stimulus, or correlates with the strength or intensity of an original affective response, is not well understood (McLaren and Mackintosh, 2002). To assess the degree to which negative affective responses to the Air Puff and Water Spray stimuli demonstrated here by the CPP test could be said to generalize, we considered the hens’ post-conditioning area use in more detail. In the CPP test, area use during tests is non-independent; avoidance of one area (the Stimulus Area) necessarily leads to increased use of other areas, but the pattern of this can vary to some extent. In the present experiments, area use differed somewhat between the two significant stimulus types, with birds that had experienced Air Puff stimuli also showing significant avoidance of the Stimulus-adjacent area (Test Day 3), but birds that had experienced Water Spray stimuli showing no generalized avoidance. However, these latter birds did show increased use of the No Stimulus-adjacent Area, the area most dissimilar to that which had become associated with the Water Spray (Test Day 1). Whether these differences signify greater generalization of negative affective responses as a result of either Air Puff or Water Spray stimulation is not clear (see Fig. 2, Fig. 3), as we would expect this to be evidenced by both of these trends. Our findings, therefore, do point to the capacity of the four chambered CPP test to gauge generalization of affective responses in a quantifiable manner, although further studies will be needed to establish whether and in what ways different stimuli affect the generalization of affective responses.

### 4.4 The Conditioned Place Preference paradigm and other tests of affect

Although the CPP test is not the only method available for detecting valenced affective states in animals, it is an important and useful one. In humans, a negatively valenced stimulus is one that we feel is unpleasant in some way and that we report disliking; across time, such stimuli can contribute significantly to both short and long-term reductions in mood and well-being and can do so in varying ways depending on the nature of the stimuli (e.g. see Fried et al., 2014). In animals, mechanisms of behavioural control vary widely, and which ones, if any, are likely to correspond to the sorts of negative feelings we experience as humans is a matter of speculation and on-going debate (e.g. see Duncan, 2002; Kirkden and Pajor, 2006; Weary et al., 2017). But even if we leave the issue of the subjective feelings to one side, it is clear that many tests do not differentiate between responses to stimuli which occur rapidly and automatically (as in Pavlovian or reflexive behavioural responses – Dolan and Dayan, 2013; Rangel et al., 2008) and those that can be said to be “affectively valenced” or “emotional” in the sense that they are under the control of the reinforcing value of the outcome (Hershberger, 1986; Mendl et al., 2010; Rolls, 2014). For example, if a preference test requires an animal to choose one resource over another by approaching it, it is not possible to say whether this preference is based on the different experienced valuations of the two (i.e. their learned reinforcing properties), or on the chosen resource triggering an approach behaviour more strongly (e.g. see O’Connell, 1979; Williams and Williams, 1969 for examples of conflict between these two processes). A preference revealed in this way could even be the result of a non-affective, habit-based response in some circumstances (e.g. as a result of over-training; Starr and Mineka, 1977; Wood and Rünger, 2016). Similarly, passive avoidance tasks, and approach-avoidance tests in which the motivational values of two stimuli are pitched against one another (e.g. highly palatable food vs noxious gas), offer indications of the scale or intensity of a given aversion, but do not clearly differentiate learned avoidance responses from more immediate behavioural responses to the reward and punisher (see Weary et al., 2017 for further discussion of the interpretation of such tests).

In sum, the CPP test is a measure of the learned association made between the reinforcement/punishment value of a stimulus and the location in which is experienced, and therefore can be said to be a useful indicator of the valenced affective state of the animal while in that situation rather than any simpler reflexive or habit-based tendency to approach or withdraw from it. And we have shown here that the basic CPP paradigm can be usefully extended to give a fuller picture of the nature of affective states induced by particular stimuli, by including information regarding the scale, persistence and generalisation of any affective responses produced. Neverthless, there remain a number of potential limitations with the use of CPP tests, including potential interference from motivational states such as hunger caused by food restriction (not used in the present studies), and issues surrounding the interpretation of chamber use in the context of different behavioural responses such as exploration and food-seeking (e.g. see Buckley et al., 2012; Dixon et al., 2013; Huston et al., 2013). While we would argue that such problems are often outweighed by the advantages of the paradigm as a whole, any interpretation of results from such studies should always be made with some degree of caution.

It is also worth noting that other tests which also make use of learned associations between stimuli and a behavioural response have potential value for measuring the multiple features of animals’ affective states, including valence, scale, persistence and generalization (Anderson and Adolphs, 2014). For example, it may be possible to modify conditioned suppression tests (Estes and Skinner, 1941) to measure facets of affect beyond valence and scale, although interpretation of such tasks is also complex, with suppressed operant responding occurring in the presence of both rewarding and aversive conditioned stimuli (Karpicke et al., 1977). And raceway or runway tests, in which animals’ learned running behaviour towards a reward is slowed by the addition of concurrent punishers, could also be developed further in these regards (e.g. see Abeyesinghe et al., 2001; Pajor et al., 2000; Rutter and Duncan, 1992).

## 5 Conclusion

The Conditioned Place Preference test is a useful measure of affective responses to potentially punishing stimuli and has value both as a method for studying the, often complex, nature of affective structures in animals, and for the assessment of stimuli potentially relevant to animal welfare.

Both air puff and water spray stimuli have been used previously as putatively aversive stimuli in a number of experiments, and our results accord with the validity of this use by demonstrating that they generate significant conditioned place aversions in hens. By demonstrating that these two stimuli generate a conditioned (i.e. learned and remembered) aversion, the present findings also indicate that such discrete stimuli can have an “affective” impact on hens – they can be said to produce negatively valenced affective states – and we conclude that such processes need to be taken into account when investigating the long-term moods and welfare of chickens (e.g. see Mendl et al., 2010).

By using a novel, four-chambered test, we have also extended the usefulness of the CPP paradigm to include measures of four fundamental facets of affective responses: valence, scale, persistence and generalization. Our findings point to the possibility that some of these facets may vary independently; for example, that stronger or more intense responses may not necessarily be more persistent, and vice versa. This too is an important possibility to be built into future studies of the impact of the effects of discrete emotive stimuli on longer-term moods and welfare, and as such is potentially relevant to the study of many species from across the animal kingdom.

## Conflicts of interest

The authors declare that there are no conflicts of interest.
